# The Placental Response to Guinea Pig Cytomegalovirus Depends Upon the Timing of Maternal Infection

**DOI:** 10.3389/fimmu.2021.686415

**Published:** 2021-06-15

**Authors:** Zachary W. Berkebile, Dira S. Putri, Juan E. Abrahante, Davis M. Seelig, Mark R. Schleiss, Craig J. Bierle

**Affiliations:** ^1^ Department of Pediatrics, Division of Pediatric Infectious Diseases, University of Minnesota, Minneapolis, MN, United States; ^2^ Informatics Institute, University of Minnesota, Minneapolis, MN, United States; ^3^ Department of Veterinary Clinical Sciences, University of Minnesota, Minneapolis, MN, United States

**Keywords:** cytomegalovirus, congenital infection, inflammation, placenta, fetal membranes, guinea pig

## Abstract

Human cytomegalovirus (HCMV) infects the placenta, and these placental infections can cause fetal injury and/or demise. The timing of maternal HCMV infection during pregnancy is a determinant of fetal outcomes, but how development affects the placenta’s susceptibility to infection, the likelihood of placental injury post-infection, and the frequency of transplacental HCMV transmission remains unclear. In this study, guinea pig cytomegalovirus (GPCMV) was used to model primary maternal infection and compare the effects of infection at two different times on the placenta. When guinea pigs were infected with GPCMV at either 21- or 35-days gestation (dGA), maternal and placental viral loads, as determined by droplet digital PCR, were not significantly affected by the timing of maternal infection. However, when the transcriptomes of gestational age-matched GPCMV-infected and control placentas were compared, significant infection-associated changes in gene expression were only observed after maternal infection at 35 dGA. Notably, transcripts associated with immune activation (e.g. *Cxcl10*, *Ido1*, *Tgtp1*, and *Tlr8*) were upregulated in the infected placenta. A GPCMV-specific *in situ* hybridization assay detected rare infected cells in the main placenta after maternal infection at either time, and maternal infection at 35 dGA also caused large areas of GPCMV-infected cells in the junctional zone. As GPCMV infection after mid-gestation is known to cause high rates of stillbirth and/or fetal growth restriction, our results suggest that the placenta becomes sensitized to infection-associated injury late in gestation, conferring an increased risk of adverse pregnancy outcomes after cytomegalovirus infection.

## Introduction

Congenital cytomegalovirus infection (cCMV), a leading cause of sensorineural hearing loss and neurocognitive disability in children, occurs in roughly 1 in 200 pregnancies (1–3). cCMV is also a cause of intrauterine growth restriction, preterm birth, and fetal demise (4–8). The timing of maternal human cytomegalovirus (HCMV) infection during pregnancy affects the rate of congenital transmission and fetal outcomes post-infection (9–18). Neurologic sequelae are most frequently observed in congenitally infected children when maternal infection occurs in the first trimester (14–17). Maternal infection late in pregnancy is associated with the highest rates of intrauterine growth restriction (IUGR) and congenital infection (9–13, 15, 18).

HCMV infects the placenta and these placental infections can cause fetal injury, including spontaneous abortion and neonatal demise, even in the absence of detectable viral transmission to the fetus (4, 5, 19–22). HCMV-associated placental pathology includes chronic villitis, cytomegalic cells, and areas of necrosis and calcification (19, 22–24). HCMV was detected in 15% of fetal remains and/or placenta after stillbirth and infection was found to be associated with fetal thrombotic vasculopathy (4). In a cohort of women with a prenatal diagnosis of fetal growth restriction, the detection of HCMV antigen in the placenta correlated with higher rates of villitis and more severe growth restriction than cases of IUGR without HCMV involvement (21). While HCMV appears to cause a hypoxia-like condition in infected placenta, how HCMV causes placental dysfunction remains poorly understood (22).

HCMV and other viruses may injure the placenta either directly by causing cytopathic effects in infected cells or by activating the maternal or fetal immune system and triggering placental immunopathology (25). Dysmature villi were frequently observed in HCMV-infected placentas, suggesting that infection can interfere with early placental development (19). Trophoblast progenitor cells can be infected by HCMV, and HCMV infection both limits the capacity of trophoblast progenitors to differentiate *in vitro* and disrupts the formation of anchoring villi in first trimester placental explants (26–28). The inflammatory response to viral infection during pregnancy can also cause placental dysfunction and adverse pregnancy outcomes (29). For example, type I interferon signaling triggered by Zika virus can cause abnormal placental development in mice and syncytial knot formation in villous explants (30). cCMV causes a proinflammatory cytokine bias in the placenta and amniotic fluid, but whether this host response disrupts normal placental function has yet to be determined (31, 32).

As the species-specificity of HCMV precludes its study in animals, guinea pig cytomegalovirus (GPCMV) has become the most widely used experimental model of cCMV (33, 34). Guinea pigs deliver precocious pups after gestational periods that average 65 days. Similarities between guinea pigs and humans include hemomonochorial placentas that invade deeply into the decidua and the prenatal development of major organs and the immune system (35–37). These similarities between human and guinea pig placentation and development make the rodent a uniquely powerful comparative model for understanding the developmental origins of health and disease (35). GPCMV infection studies have revealed that placental and fetal infection occur sequentially after maternal inoculation (34, 38–41). If maternal infection occurs early in pregnancy, infectious virus may not be detected in pup organs and evidence of prior fetal infection can be limited to the presence of infection-associated histologic lesions in pup organs (41–43). Maternal GPCMV infection after mid-gestation has been found to cause high rates of stillbirth and IUGR in pups (42–44).

In this study, we investigated whether the susceptibility and antiviral responses of the placenta to GPCMV infection varied across gestation. Time mated guinea pigs were infected either at 21 days gestation (dGA, “early”) or at 35 dGA (“late”). No significant differences in maternal or placental viral loads were noted between the two groups at 21 days post-infection (dpi). However, infection after mid-gestation caused more frequent fetal membrane infections and significant changes in placental gene expression that were not observed after infection at the earlier time point. Furthermore, *in situ* hybridization revealed that GPCMV infection primarily localized to the junctional zone after maternal infection at the later time. Our observations lead us to propose that GPCMV-associated stillbirth and IUGR are the result of developmentally regulated changes in the placenta that predispose the organ to infection-associated injury late in gestation.

## Materials and Methods

### Cells and Virus

A minimally tissue culture adapted stock of GPCMV 22122 (ATCC VR-682), prepared as previously described by passaging guinea pig salivary gland homogenate twice on JH4 guinea pig lung fibroblasts (ATCC CCL-158), was used for this study (33, 45, 46). JH4 cells were purchased from ATCC and propagated according to their specifications excepting that the growth media was supplemented with sodium bicarbonate (47). GPCMV stocks were prepared as previously described and titered on JH4 cells (48). For animal studies, virus was aliquoted into single-use units, flash-frozen, and stored at -80°C. These aliquots were routinely re-titered as guinea pigs were infected to confirm that the stock did not deteriorate during storage.

### Ethics Statement

All animal procedures were conducted in accordance with protocols approved by the Institutional Animal Care and Use Committee (IACUC) at the University of Minnesota, Minneapolis (Protocol ID: 1810-36403A). Experimental protocols and endpoints were developed in strict accordance to the National Institutes of Health Office of Laboratory Animal Welfare (Animal Welfare Assurance #A3456-01), Public Health Service Policy on Humane Care and Use of Laboratory Animals, and United States Department of Agriculture Animal Welfare Act guidelines and regulations (USDA Registration # 41-R-0005) with the oversight and approval of the IACUC. Outbred Hartley guinea pigs were initially purchased from Elm Hill Laboratories (Chelmsford, MA). Breeding pairs of strain 2 and strain 13 guinea pigs were generously shared by MS and the U.S. Army Medical Research Institute of Infectious Diseases, respectively. Guinea pigs were housed in a facility maintained by the University of Minnesota Research Animal Resources, who were accredited through the Association for Assessment and Accreditation of Laboratory Animal Care, International (AAALAC). All procedures were conducted by trained personnel under the supervision of veterinary staff.

### Animal Pathogenicity Study

The GPCMV serostatus of all animals received from an external source was tested by ELISA within one week of receipt and again after one month of housing at the University of Minnesota (49). Only GPCMV seronegative animals were used for infection studies. Female guinea pigs were bred at two to three months of age. After the delivery of their first litter, the animals were bred a second time during postpartum estrus to establish timed pregnancies by housing with a male for three days postpartum. These second pregnancies were confirmed by progesterone ELISA (DRG International); only animals with plasma progesterone concentrations exceeding 15 ng/ml by 20 days postpartum were included in this study (50).

At either 21 (range 18 to 23) or 35 (range 33 to 35) dGA guinea pigs were injected subcutaneously into the scruff of the neck with 0.5 ml of PBS containing 2x10^5^ PFU of GPCMV or PBS alone. Blood and plasma were collected from dams every seven days post-infection (dpi) until they were euthanized at 14, 21, or 28 dpi. After euthanasia, blood and plasma were collected from the dams and amniotic fluid was collected from the pups. Pup tissue samples were divided and frozen for DNA extraction, stabilized in RNAlater (ThermoFisher) for RNA extraction, embedded in optimal cutting temperature (O.C.T.) compound (Fisher Scientific), or immersion-fixed using Shandon Formal-Fixx (ThermoFisher) and embedded in paraffin.

### Viral Load Quantification by Droplet Digital PCR

After DNA was extracted from whole blood, tissue, and amniotic fluid, GPCMV genomes were quantified by droplet digital PCR (ddPCR) using primers and probes specific to *GP54* using the Bio-Rad QX200 system as previously described (46). ddPCR results were analyzed using the QuantaSoft™ Analysis Pro software (Bio-Rad); GPCMV viral loads are presented as the number of copies genome per ml of fluid or mg of tissue.

### RNA Sequencing

RNA was extracted from guinea pig placentas that had been stabilized in RNAlater (ThermoFisher) and stored at -20°C using the RNAeasy Mini Kit (Qiagen). ~30 mg pieces of placenta were combined with 0.6 ml of β-mercaptoethanol-containing RLT buffer and Lysing Matrix D (MP Biomedicals) and pulsed at 6 m/s for 30 seconds in a FastPrep 24 (MP Biomedicals). RNA was extracted from 0.45 ml of the resulting homogenate using the manufacturer’s standard protocol, including the optional on-column DNase I digest (Qiagen), Total RNA integrity was assessed by capillary electrophoresis using an Agilent TapeStation; all samples used for library creation had RNA integrity numbers that exceeded 7.0.

Dual indexed TruSeq stranded mRNA libraries were prepared and sequenced using a NextSeq 550 high-output 75-bp single-end run (mean of 22.4x10^6^ reads/sample). FastQ reads were trimmed using Trimmomatic (v 0.33) enabled with the optional “-q” option; 3bp sliding-window trimming from 3’ end requiring minimum Q30 (51). Quality control on raw sequence data for each sample was performed with FastQC. Read mapping was performed *via* Hisat2 (v2.1.0) using the “Cavpor3.0” genome (GCF_000151735.1) as reference (52). Gene quantification was done *via* Cuffquant for FPKM values and Feature Counts for raw read counts (53, 54). Differentially expressed genes were identified using the edgeR (negative binomial) feature in CLCGWB (Qiagen) using raw read counts (55). The generated list was filtered based on a minimum 2X Absolute Fold Change and False Discovery Rate corrected *p* < 0.05. Raw and processed RNA-Seq data have been deposited in NCBI’s Gene Expression Omnibus and are accessible through GEO Series accession number GSE169358 (56). Normalized gene expression data (FPKM) was uploaded to ClustVis for data visualization and heat map analysis (57). Gene Ontology (GO) analysis was performed by uploading a list of differentially expressed transcripts to g:Profiler (58). Results from this GO analysis were visualized using Cytoscape, Enrichment Map, ClusterMaker2, and WordCloud (59–63).

### Real-Time Droplet Digital PCR Analyses

RNA was extracted from placenta as described above. A two-step reverse transcriptase ddPCR (RT-ddPCR) protocol was used to quantify transcript abundance (46). cDNA was synthesized from total RNA using the Maxima™ H Minus cDNA Synthesis Master Mix (ThermoFisher). PCR primers targeting guinea pig transcripts were designed using the Primer Quest tool and were ordered from Integrated DNA Technologies (**Table S2**). ddPCR reactions were prepared using the EvaGreen Digital PCR Supermix (Bio-Rad) using a primer concentration of 250 nM and cycled using the following thermal conditions: 95°C for 5 min; 40 cycles of 95°C for 30 s and 60°C for 30s; 4°C for 5 min; 90°C for 5 min; hold at 4°C. The ddPCR data were analyzed with QuantaSoft™ Analysis Pro software (Bio-Rad). Absolute quantification of gene expression was presented as copies per nanogram of total RNA.

### *In Situ* Hybridization

5-μm sections of paraffin-embedded placenta were mounted onto Superfrost Plus slides (ThermoFisher). After air drying the tissue sections overnight, the slides were baked at 60°C for 1 hr. Tissue was deparaffinized and pretreated using the recommended protocol for RNAscope^®^ 2.5 Assays (ACD Document #322452). For target retrieval, samples were incubated at 99°C for 15 minutes, and the slides were treated with RNAscope Protease Plus for 30 minutes. Slides were stained using either the RNAscope 2.5 HD Detection Reagent – RED (ACD Document # 322360-USM) or the RNAscope 2.5 HD Duplex Reagent (322500-USM) and the RNAscope Probe V-CavHV-2-gp3. Stained slides were scanned using a Huron TissueScope LE and the number of *gp3+* foci and areas of *gp3*-staining were counted and calculated using NIS-Elements BR (Nikon).

## Results

### The Timing of Maternal GPCMV Infection Affects the Rate of Fetal Membrane Infection but Not Placental Viral Loads

**Figure 1:**
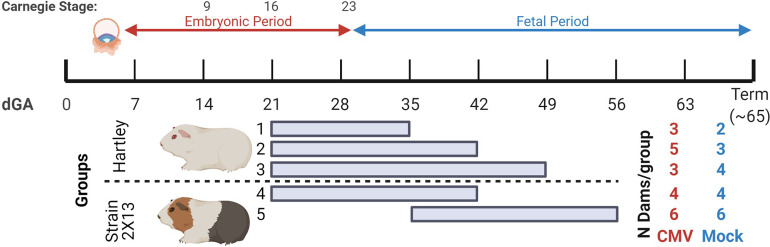
Summary of GPCMV infections in time mated guinea pigs. The ~65-day gestational period of guinea pigs is diagramed. Implantation occurs at 5-6 dGA and the embryonic period ends at 29 dGA. Reference Carnegie stages for guinea pig development are indicated (64). Five groups of GPCMV-infected and control guinea pigs were used in this study, including outbred Hartley guinea pigs and inbred strain 2 females that were bred to strain 13 males (producing semi-allogenic, hybrid pregnancies). Guinea pigs were infected either at 21 dGA (embryonic period) or at 35 dGA (fetal period). The number of GPCMV- and mock-infected dams for each group is shown. Figure created with BioRender.com.

To study how the timing of maternal GPCMV infection affects viral loads in the placenta, extraplacental membranes, and fetus, guinea pigs were time mated during postpartum estrus and infected at either 21 or 35 days gestation (dGA) (**Figure 1**). HCMV infection during the first trimester causes most cases of neurologic disability in congenitally infected children, and infecting guinea pigs at 21 dGA exposes the pup and placenta to virus during a comparable period of late embryonic/early fetal development (14–17, 65). GPCMV infection after mid-gestation has been found to often cause fetal growth restriction and/or stillbirth (42–44). As high rates of ischemic injury and virus-specific focal necrosis and inflammation had been reported 21 days after maternal infection at 30 dGA, we elected to infect animals at 35 dGA and analyze matched pups and placentas at 21 dpi, before expected still- or preterm birth, to identify mechanisms that could cause these adverse pregnancy outcomes (39).

Five groups of animals were used in this study. Three groups of Hartley guinea pigs were infected at 21 dGA and euthanized at either 14, 21, or 28 dpi. The remaining two groups were inbred strain 2 females bred with strain 13 males to create hybrid pregnancies (2X13) and were infected with GPCMV at either at 21 or 35 dGA and euthanized at 21 dpi (66). This study compared GPCMV pathogenesis between the outbred and inbred guinea pigs and examined the effect of maternal infection at the two different times on the placenta. Most prior GPCMV research has been done in Hartley guinea pigs, and the few studies that compared GPCMV infection between inbred and outbred guinea pigs have yielded conflicting results as to whether strain 2 or strain 13 animals are more susceptible to infection than Hartley guinea pigs (67–69).

Guinea pigs were infected with 2X10^5^ PFU of GPCMV or mock-infected by subcutaneous injection. While Hartley dams were on-average larger than strain 2 females, no differences in maternal weight gain were noted between GPCMV-infected and control dams (**Figure S1**). Maternal blood and serum were collected weekly until each animal’s predetermined endpoint. GPCMV viral load was quantified using a droplet digital PCR (ddPCR) assay targeting *GP54* (46). When maternal viremia at 7 dpi and viral loads in spleen were quantified, infection was confirmed in all dams and no significant differences were noted between the five groups (**Table S1** and **Figure S2A**).

**Figure 2:**
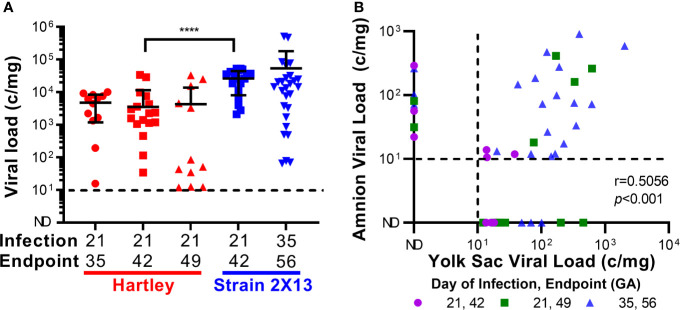
GPCMV viral loads after maternal infection during pregnancy. Time mated guinea pigs were infected at either 21 or 35 dGA with 2X10^5^ PFU of GPCMV and euthanized 14, 21, or 28 days later. DNA was extracted from tissues and GPCMV viral loads were determined using a ddPCR assay specific to *GP54.* The limit of detection for this assay is indicated by the dashed line. **(A)** Viral load in placentas. Significantly higher viral loads (Mann-Whitney test, **** *p <* 0.0001) were observed in the placentas of strain 2X13 hybrids than in the placentas of Hartley guinea pigs. **(B)** Viral loads in infected fetal membranes. The number of GPCMV genome copies detected in the amnion and visceral yolk sac of individual fetuses was significantly correlated (Pearson r=0.5056, *p <* 0.001).

The placentas from the two groups of guinea pigs that were infected at 21 dGA and euthanized at 21 dpi were compared, and significantly higher viral loads were detected in the strain 2X13 placentas than in their Hartley counterparts (Mann–Whitney U test, P<0.0001) (**Figure 2A**). No other significant differences in placental viral load were noted in the remaining groups. When viral loads were assessed in the extraplacental membranes, GPCMV was detected in both the amnion and visceral yolk sac (**Table 1** and **Figure S2B**) (46). A significant correlation (Pearson r=0.5056, *p*<0.001) in GPCMV viral loads was observed in the amnion and yolk sac of individual fetuses (**Figure 2B**). The highest frequencies of fetal membrane infection and the highest viral loads in the membranes were observed in the dams infected at 35 dGA. GPCMV was only detected in a handful of amniotic fluid samples collected from guinea pigs infected at 35 dGA, consistent with previous observations that GPCMV generally does not accumulate in amniotic fluid (43, 46, 69).

**Table 1:** Summary of GPCMV viral load data.

Strain	dGA of infection, endpoint	Placenta		Amnion		Yolk Sac		Fetus^3^	AF^4^
		CMV+^1^	MVL^2^	CMV+^1^	MVL^2^	CMV+^1^	MVL^2^	CMV+^1^	CMV+^1^
**Hartley**	21,35	12/12	5.7x10^3^	0/10	N/A	0/10	N/A	9/10	0/10
	21,42	17/29	6.0x10^3^	4/29	1.5x10^1^	4/28	2.0x10^1^	9/29	0/29
	21,49	12/19	6.8x10^3^	6/19	1.6x10^2^	11/19	1.8x10^2^	9/19	0/18
**2X13**	21,42	18/19	2.8x10^4^	2/18	1.7x10^2^	2/18	1.7x10^1^	0/18	0/18
	35,56	27/27	5.4x10^4^	18/24	1.8x10^2^	22/24	2.5x10^2^	4/27	4/21

^1^Samples containing detectable GPCMV DNA/total number of samples.

^2^Mean viral load (MVL) expressed as copies/ml for infected blood and amniotic fluid or copies/mg for infected tissue.

^3^Viral load in fetal brain quantified.

^4^Amniotic fluid.

Fetal infection was assayed by quantifying viral loads in the brain. GPCMV was most often detected at 14 dpi and occasionally at later times post-infection. As the brain is less frequently infected by GPCMV than other fetal tissues, these results may underreport the actual rate of congenital GPCMV infection in this experiment and/or indicate that fetal infections resolved *in utero* (42, 43). The fetuses of GPCMV infected dams trended smaller than their mock-infected counterparts in most cases (**Table S1** and **Figure S2C**). However, due in part to unexpectedly large variation in litter sizes, this study was underpowered to determine whether GPCMV caused fetal growth restriction. Guinea pigs bred during postpartum estrus had significantly larger litters than expected, including many large litters of 6-8 pups, and fetal weights correlate with litter size after mid-gestation (37).

To summarize, our animal studies found that maternal infection at 35 dGA resulted in higher rates of fetal membrane infection and higher viral loads in the amnion and yolk sac than infection at 21 dGA. However, neither the timing of maternal infection nor the experimental endpoint had a significant effect on placental viral load. Higher viral loads were noted in placentas from our strain 2X13 hybrid pregnancies when compared to Hartley guinea pigs, but there was no other evidence that indicated that infection was more severe in the inbred animals. Presuming that there would be minimal animal-to-animal variation in the placental response to infection in guinea pigs with a consistent maternal and fetal genetic background, we focused on the strain 2X13 animals in our subsequent analysis of the effect of infection on placental function.

### GPCMV Infection After Mid-Gestation Significantly Alters Placental Gene Expression

**Figure 3:**
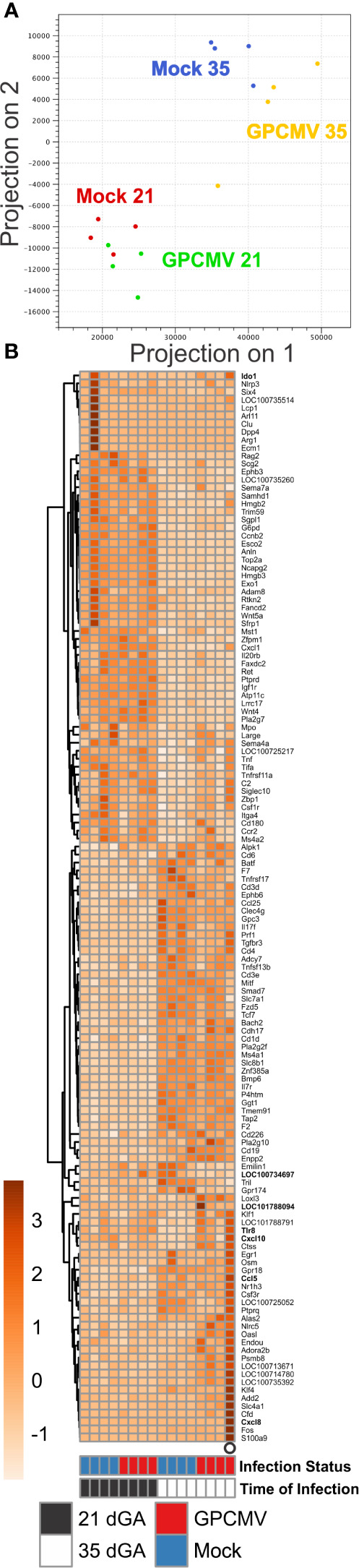
Transcriptome profiling of GPCMV-infected placenta. Time mated guinea pigs were GPCMV- or mock-infected at 21 or 35 dGA and euthanized 21 days later. RNA was extracted from placentas (N=4/group) and gene expression was quantified by Illumina RNA-Seq. **(A)** Principal component analysis illustrating the similarity in gene expression between samples. **(B)** Heat map illustrating the relative expression of transcripts that function as part of an immune system processes (GO:0002376) that were differentially expressed in placenta either during normal development or after GPCMV infection. Transcripts that were also analyzed by RT-ddPCR are shown in bold.

Having found that the timing of maternal GPCMV infection did not affect placental viral loads, we next compared how infection at our earlier and later time point affected placental gene expression. For this analysis, placentas were randomly selected from inbred guinea pigs that had been GPCMV- or mock-infected either at 21 or 35 dGA and euthanized at 21 dpi. RNA was extracted from four placentas from four GPCMV-infected dams and four placentas from three control dams per group and sequenced (**Table S1**). A principal component analysis of RNA-Seq data revealed that the samples clustered based upon the gestational age of placenta (**Figure 3A**). GPCMV- infected and control placenta from the early infection groups clustered tightly, while there was clear separation between GPCMV- and mock-infected samples after maternal infection at 35 dGA. Pairwise gene expression comparisons between age-matched groups of GPCMV and mock-infected tissues affirmed these findings. GPCMV infection at 21 dGA had a limited effect on placental gene expression at 21 dpi: only 8 transcripts were differentially regulated (≥2 fold, *p* < 0.05). In contrast, maternal infection at 35 dGA resulted in the differential regulation of 126 transcripts (≥2 fold, *p* < 0.05) at 21 dpi. A gene set enrichment analysis was performed on the transcripts that were differentially expressed after GPCMV infection late in pregnancy (58). This analysis found that several gene ontology terms related to the immune response (including GO:0002376, immune system process) were significantly enriched after GPCMV infection at 35 dGA (**Figure S3A**).

In a pairwise gene expression analysis that compared the two groups of mock-infected placentas, the gestational age of placentas was found to have a much more significant effect on gene expression than GPCMV infection: 1438 transcripts were differentially regulated (≥2 fold, *p* < 0.05) between the two groups of control tissue. Gene ontogeny analysis found numerous terms related to the regulation of the mitotic cell, signaling receptor activity, and condensed chromosome regions were enriched in this pairwise comparison (**Figure S3B**). Many of the gene expression differences between normal placenta from 42 and 56 dGA appear to be related to the relatively higher expression of transcripts involved in cell division and the cell cycle by the younger tissue. While transcripts that function as part of an immune system process were not found to be significantly enriched in our comparison of normal placentas, 116 transcripts related to the immune response were differentially expressed between the two groups (**Figure 3B**, **Table S3**). We hypothesize that these normal changes in placental immunity may cause GPCMV infection after mid-gestation to significantly affect placental gene expression.

**Figure 4:**
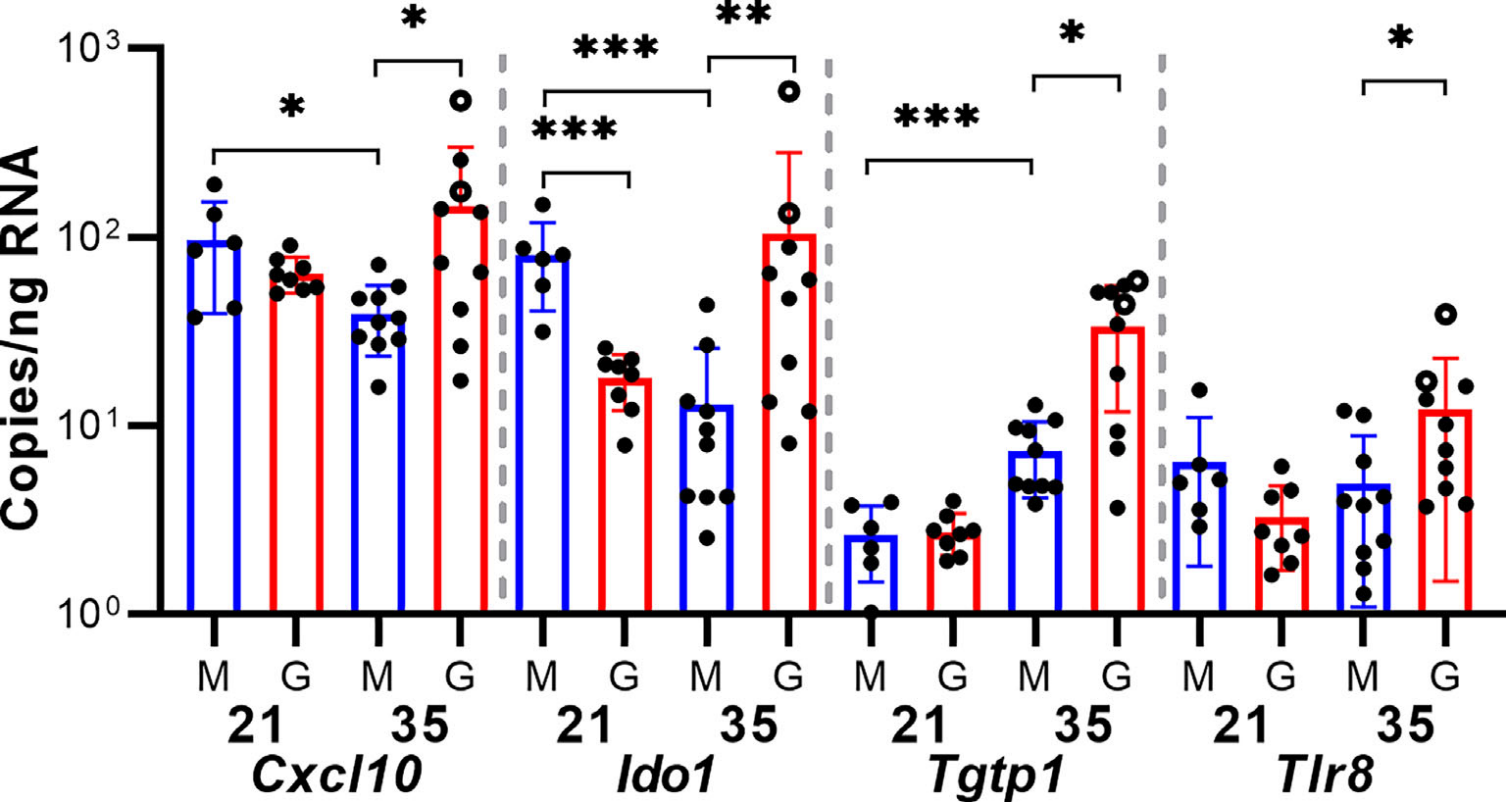
GPCMV infection dysregulates immune gene transcription. A two-step RT-ddPCR protocol was used to quantify the expression of select transcripts in GPCMV- (G) and mock-infected (M) placentas after maternal infection at either 21 or 35 dGA. Data from two placentas per dam is shown. Statistically significant differences were calculated by Mann-Whitney test (*p < 0.05, **p < 0.01, ***p < 0.001).

As only four samples of placenta per group were analyzed by RNA-seq, sample-to-sample variation in gene expression could be caused either by regional differences in transcription in the relatively large guinea pig placenta or represent the unique responses of individual fetuses to GPCMV infection. To better elucidate host factors that regulate the placenta’s response to GPCMV, we used reverse transcriptase droplet digital PCR (RT-ddPCR) to measure the expression of select transcripts that function as part of the inflammatory response in additional placenta samples. For this experiment, RNA was extracted from two placentas per dam (including the samples that had been previously analyzed by RNA-Seq). RT-ddPCR confirmed that four genes– *Cxcl10*, *Ido1*, *Tgtp1*, and *Tlr8*–were significantly upregulated after maternal infection at 35 dGA when compared to age-matched control placentas (**Figure 4**). This analysis also found that *Cxcl10* and *Ido1* are normally downregulated as the guinea pig placenta matures and that GPCMV infection at 21 dGA leads to decreased *Ido1* expression relative to age-matched normal placenta. RT-ddPCR analysis did not support the RNA-Seq finding that several other inflammatory mediators—*Ccl5*, *Ccl15-l*, *Cxcl8*, *Il1b*, and *Il36b-l*—were differentially regulated by maternal GPCMV infection at 35 dGA (**Figure S4**). In the case of *Cxcl8* and *Il1b*, high levels of cytokine transcription were noted in the placenta of a dam that had been euthanized while delivering stillborn pups at 56 dGA (these placentas are represented as open circles in **Figures 3**, **4**, and **Figure S4**). The elevated transcription of these cytokines may not be specific to GPCMV infection and instead be inflammatory markers of preterm labor or *in utero* fetal demise (70, 71). Cumulatively, our gene expression analyses of GPCMV-infected placenta suggest that the immune response is dysregulated by GPCMV infection after mid-gestation but not after infection earlier in pregnancy.

### The Junctional Zone Becomes Infected by GPCMV After Maternal Infection After Mid-Gestation

**Figure 5:**
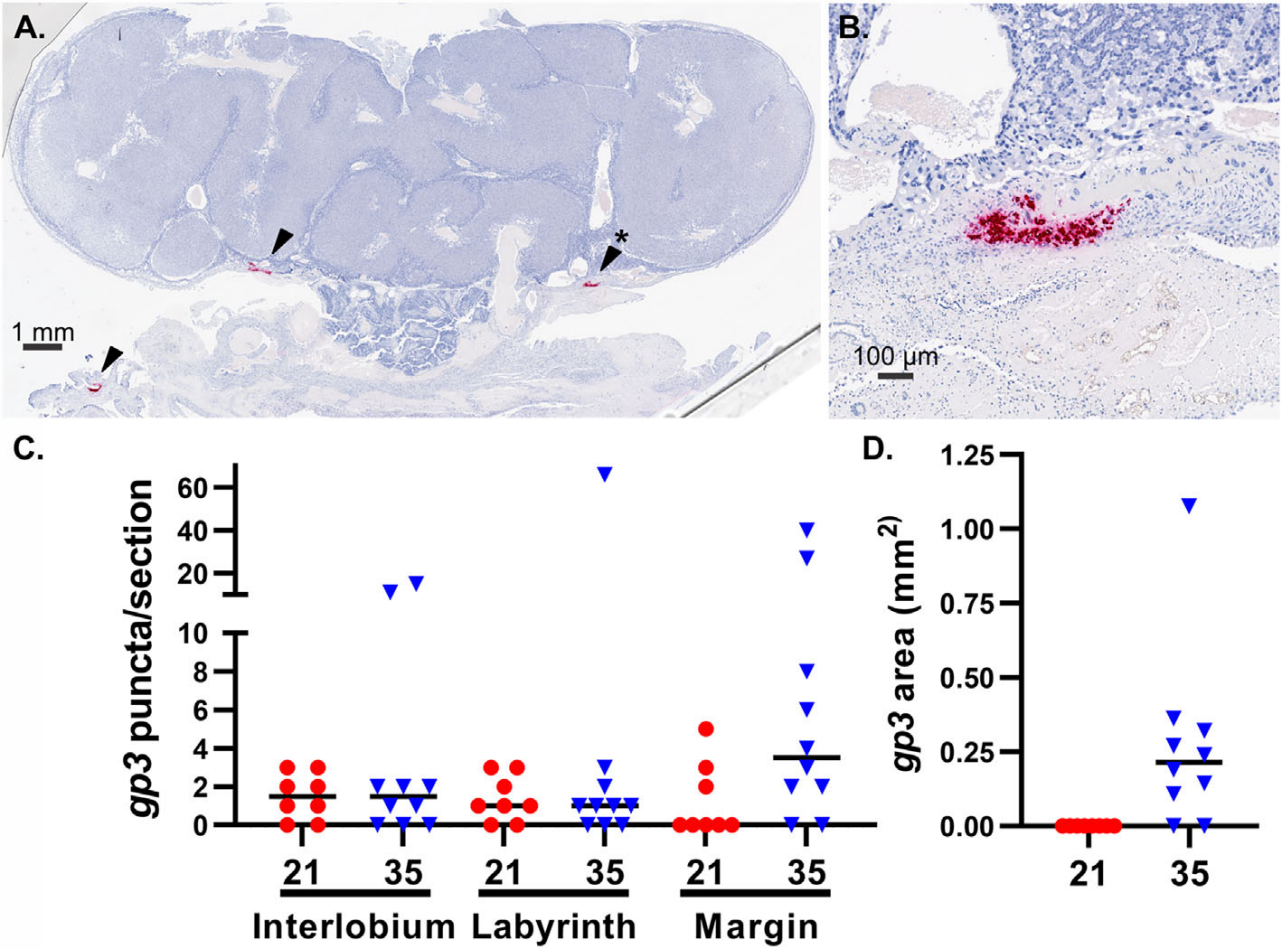
GPCMV localization in infected placenta. An RNAscope probe specific to GPCMV *gp3* was used to detect infected cells in sections of placenta by *in situ* hybridization. **(A)** A representative section of a placenta from a dam infected at 35 dGA, illustrating large areas of GPCMV infected cells in the junctional zone and decidua (red, indicated by arrows). **(B)** High-magnification image of infected cells in the junctional zone (*). **(C)** *gp3*+ puncta, representing individual infected cells or small groups of infected cells, were counted based on their localization in the placenta after maternal infection at 21 or 35 dGA and collection at 21 dpi. **(D)** The measured area of *gp3*+ stained cells in the junctional zone of placentas is shown.

Finally, we compared the frequency and localization of GPCMV-infected cells in the placenta using *in situ* hybridization. For this study, we designed an RNAscope probe specific to the GPCMV transcript *gp3*. RNA-Seq analysis had revealed that *gp3* is highly expressed during all phases of GPCMV replication (**Figure S5**) (46). *gp3+* foci, each representing either an individual infected cell or a group of infected cells, were manually counted in stained placenta sections (**Figure 5**). A small number of *gp3*+ foci were observed in the interlobium and labyrinth of all GPCMV-infected placenta (**Figure 5C**). When the placentas of dams infected at 21 and 35 dGA were compared, significantly more GPCMV infected cells were noted in the margin of placentas, including either the parietal yolk sac or marginal syncytium, after infection at the later time. Large areas of *gp3+* cells were detected in the junctional zone in eight of ten of the placentas from the late infection group (**Figures 5A, B, D**). These large areas of GPCMV-infected cells were often adjacent to either the subplacenta or large blood vessels and were never detected after maternal infection at 21 dGA. Whether GPCMV infection of the junctional zone reflects the recruitment of infected cells to the placenta late in pregnancy or if there is a cell type in this region that becomes permissive to GPCMV infection as the placenta matures remains to be determined.

## Discussion

The timing of HCMV infection during pregnancy is a determinant of fetal outcomes, yet how placental development affects the course and severity of cCMV remains poorly understood (25, 72). Using the GPCMV model, we assessed how the placenta and fetal membranes are differentially affected by maternal infection at two times: 21 and 35 dGA. Prior research has found that GPCMV infection early in pregnancy can resolve prenatally while infection after mid-gestation often causes stillbirth and fetal growth restriction (42–44). Our study identified several possible explanations for these adverse pregnancy outcomes, including an increased rate of fetal membrane and junctional zone infection late in gestation and a transcriptional response to GPCMV that may indicate that placental immunopathology only occurs after maternal infection at later times in pregnancy.

This study found that GPCMV infects both the amnion and the yolk sack, that viral loads in the two membranes are correlated, and that the highest rates of infection and viral loads in the membranes occur after maternal infection late in pregnancy (46). While GPCMV joins HCMV on a short list of viruses that have been observed to infect the fetal membranes, it remains unclear whether or how viral infections of the fetal membrane affect the fetus (24, 73). In contrast, ascending bacterial infections, a significant cause of preterm labor, have a much better understood effect on fetal membrane physiology. Bacterial infection, treatment with inflammatory cytokines, or exposure to pathogen-associated molecular patterns can all trigger preterm labor in animal models and/or cause fetal membrane explants to biomechanically weaken *ex vivo* (71, 74–76). Several studies have suggested that viral infection may sensitize the fetal membranes to later damage from bacteria (77–79). It has also been hypothesized that fetal membrane infection may allow viruses to circumvent the potent antiviral defense of the placenta to infect the fetus (80). In this paraplacental route of infection, viruses that infect the chorioamnion may be shed into amniotic fluid and infect the fetus. Given that this study’s primary focus was on the effect of infection on the placenta, future experiments that specifically analyze the effect of GPCMV on the fetal membranes are merited.

Developmentally programmed changes in maternal, placental, and fetal immunity are critical for healthy pregnancy, helping to establish and maintain maternal-fetal tolerance, remodel the uterus, and regulate parturition (30, 71). Alterations in maternal immunity can increase the severity of viral infections late in pregnancy (81). Severe, third-trimester infections caused by viruses such as influenza and hepatitis E can cause fetal injury or demise. High rates of maternal demise have been reported in some GPCMV infection studies (38, 82). However, HCMV infections are typically mild and there is no evidence that the virus causes more severe illness in pregnant individuals (83). We found that the timing of maternal GPCMV infection did not significantly affect maternal, placental, or fetal viral loads, and severe illness was not noted in dams that had been infected at the later time. Having eliminated severe maternal illnesses as a cause of adverse pregnancy outcomes in our experiments, the remainder of this study focused on the placental response to GPCMV infection.

Prior work in placental cells and explants has found that gestational age affects how permissive the cells or tissue are to viral infection and the nature of the inflammatory response that is triggered by infection (84, 85). Our study found that maternal infection at 35 dGA significantly altered placental gene expression while infection at 21 dGA had remarkably little effect on placental gene expression. Infection after mid-gestation upregulated numerous transcripts that function as part of an immune system process. Two transcripts that are dysregulated by placental GPCMV infection, *Cxcl10* and *Ido1*, are particularly relevant to the pathogenesis of cCMV. CXCL10, a biomarker of VUE, chronic chorioamnionitis, and late preterm birth, is found at elevated concentrations in amniotic fluid and maternal sera during cCMV (31, 86–89). Curiously, while the concentration of CXCL10 in amniotic fluid correlates with CMV genome abundance, the chemokine does not appear to accumulate in amniotic fluid when other viruses cause intrauterine infections (32). We and others have found that CXCL10 transcription is upregulated when placental cells or tissue are infected with HCMV or GPCMV *in vitro* (46, 84, 85). Indoleamine-2,3-dioxygenase (IDO) is an enzyme that is encoded by two genes, *IDO1* and *IDO2*, that catalyze the rate-limiting step in tryptophan catabolism. IDO production by the placenta and the subsequent metabolism of tryptophan prevents the rejection of the allogeneic fetus by suppressing T cell proliferation and activity (90). IDO is more highly expressed by the human placenta during the first-trimester than at term and HCMV infection suppressed IDO expression in early placenta (91). While our gene expression analysis was limited to a single time point post-infection, GPCMV may cause placental immunopathology but only after maternal infection relatively late in pregnancy.

In an analysis of placental pathology after GPCMV infection, pregnant Hartley guinea pigs were inoculated with GPCMV at 30 dGA. Infection caused ischemic injury and necrosis associated with acute or chronic inflammation beginning at 14 dpi and GPCMV-specific antigens and viral particles were most frequently observed at 28 dpi in the marginal and interlobar transitional zones of the main placenta (39). We used *in situ* hybridization to compare the localization of GPCMV in the placenta 21 days after infection at 21 or 35 dGA. Like Griffith and colleagues, we detected occasional infected cells in the main placenta after infection at either time. However, the most consistent pattern of infection and largest lesions localized to the junctional zone, which is situated between the main placenta and the maternal decidua. These lesions were only found after infection at 35 dGA and may indicate that a ring of GPCMV-sensitive cells develop at the base of the placenta as term approaches. Given how frequently we detected this pattern of junctional zone infection, we were surprised it had not been previously reported. However, because our RNAscope assay targets a viral transcript (*gp3*) that is highly expressed during all phases of GPCMV’s replication, *in situ* hybridization may be more sensitive than electron microscopy or immunohistochemistry for detecting GPCMV infected cells.

Cumulatively, our data suggests the placenta becomes more sensitized to GPCMV infection-associated injury late in gestation either due to a potentially pathogenic immune response or by the fetal membranes and junctional zone becoming more permissive to infection. Numerous questions remain. Because GPCMV-associated lesions had previously been reported exclusively in the main placenta, we extracted DNA and RNA for viral load quantification and gene expression analyses from this tissue (37, 39). A more nuanced analysis that compares the rate of GPCMV infection and the immune response in the decidua, junctional zone, and main placenta is justified. Our study did not investigate the effects of infection during the earliest stages of pregnancy. One report has noted that maternal GPCMV infection immediately prior to conception caused high rates of fetal demise, suggesting that the virus may perturb early placental development and function (40). Due to the limited availability of normal tissue between twenty weeks gestation and term, how viral infection effects the function of the human placenta after mid-gestation remains poorly characterized. Given similarities in the placental response of humans and guinea pigs to CMV infection and that comparative gene expression analyses suggest that murine placental development largely parallels the first half of human pregnancy, guinea pigs may be particularly well suited to model intrauterine infection after mid-gestation (92).

## Data Availability Statement

The datasets presented in this study can be found in online repositories. The names of the repository/repositories and accession number(s) can be found in the article/**Supplementary Material**.

## Ethics Statement

The animal study was reviewed and approved by Institutional Animal Care and Use Committee at the University of Minnesota.

## Author Contributions

CB conceived and designed the study. CB, ZB, and DP collected the data. All authors participated in data analysis and interpretation. CB drafted the manuscript. All authors contributed to the article and approved the submitted version.

## Funding

This project was supported by grants from the Minnesota Masonic Charities (Masonic Early Investigator Award) and the National Institutes of Health (R21HD087496, R01HD098866, and UL1TR002494).

## Conflict of Interest

The authors declare that the research was conducted in the absence of any commercial or financial relationships that could be construed as a potential conflict of interest.
