# Supplementary figures and images for: The Placental Response to Guinea Pig Cytomegalovirus Depends Upon the Timing of Maternal Infection
Source: Front Immunol. 2021 Jun 15;12:686415. doi: 10.3389/fimmu.2021.686415 (PMC8239309; doi:10.3389/fimmu.2021.686415)

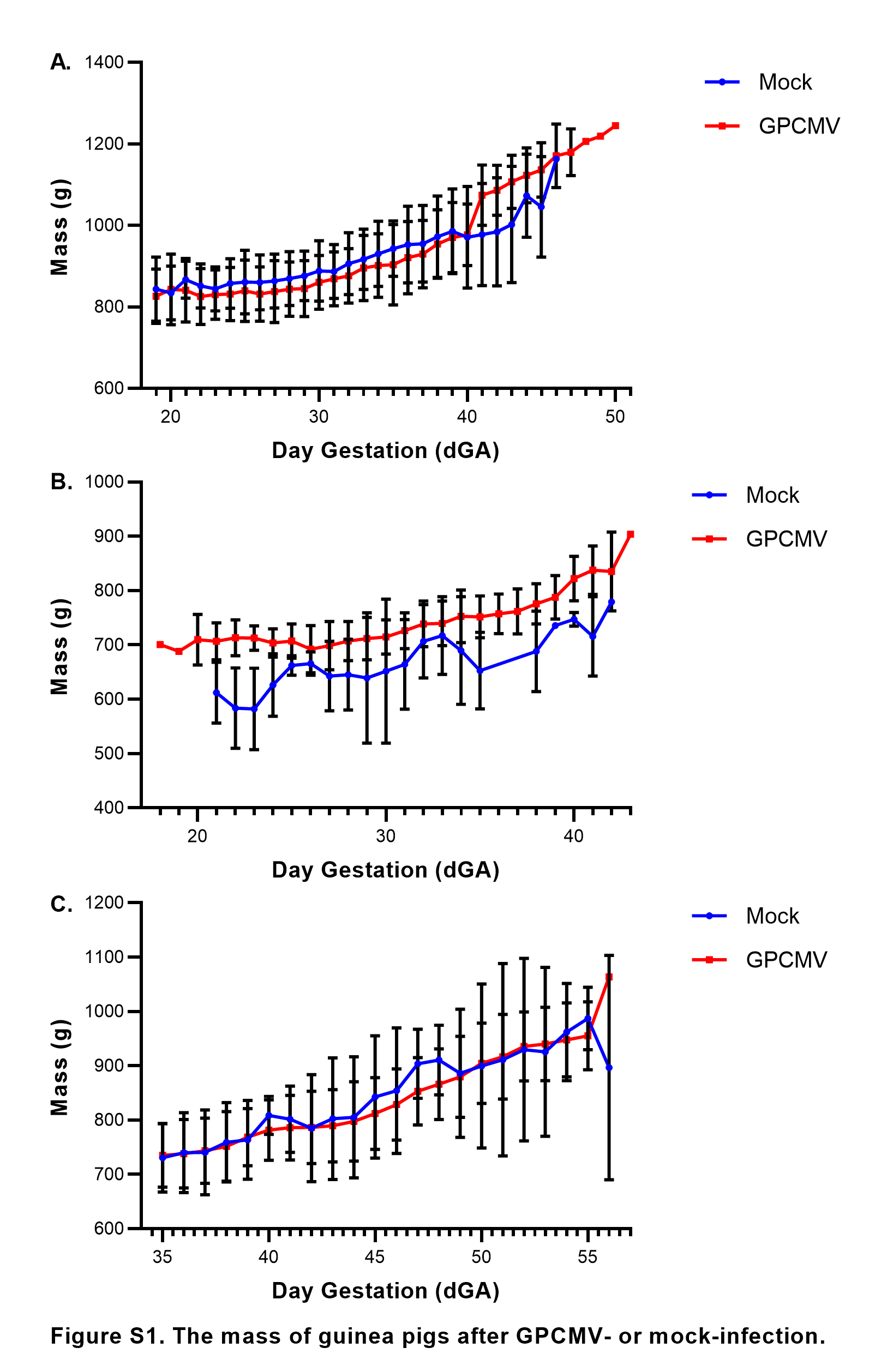

Supplement: Supplementary file 1 [file Image_1.tif]

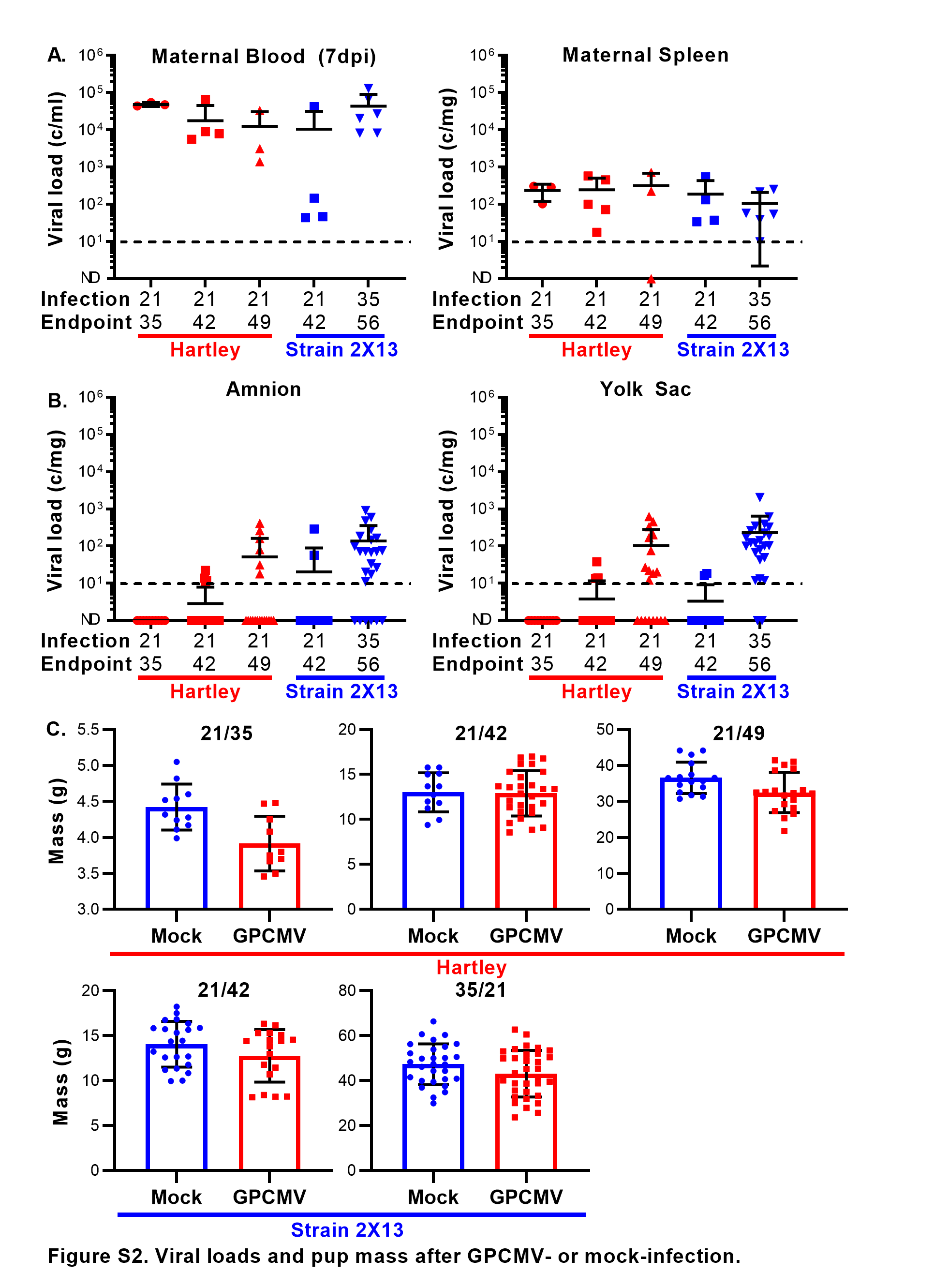

Supplement: Supplementary file 2 [file Image_2.tif]

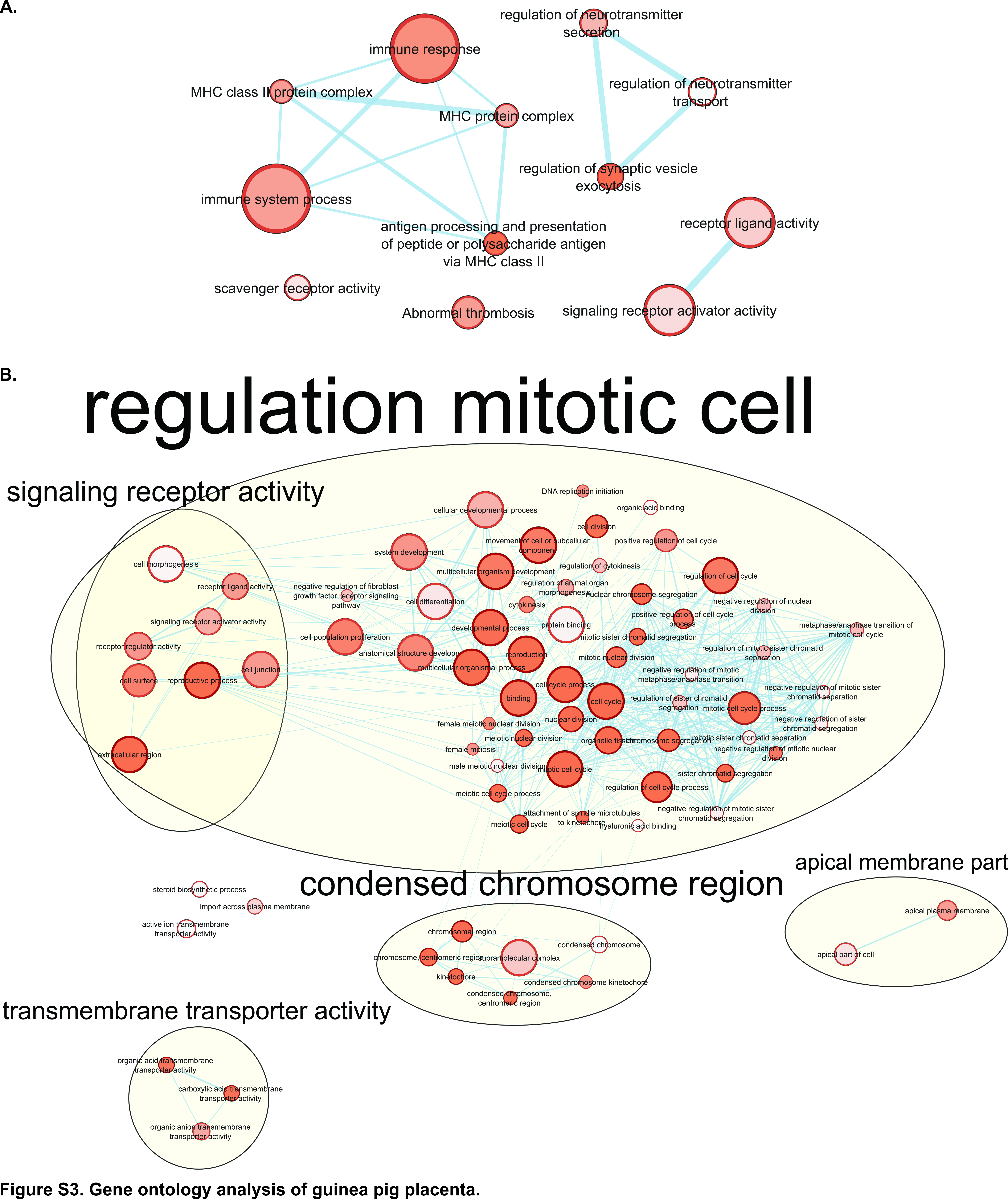

Supplement: Supplementary file 3 [file Image_3.tif]

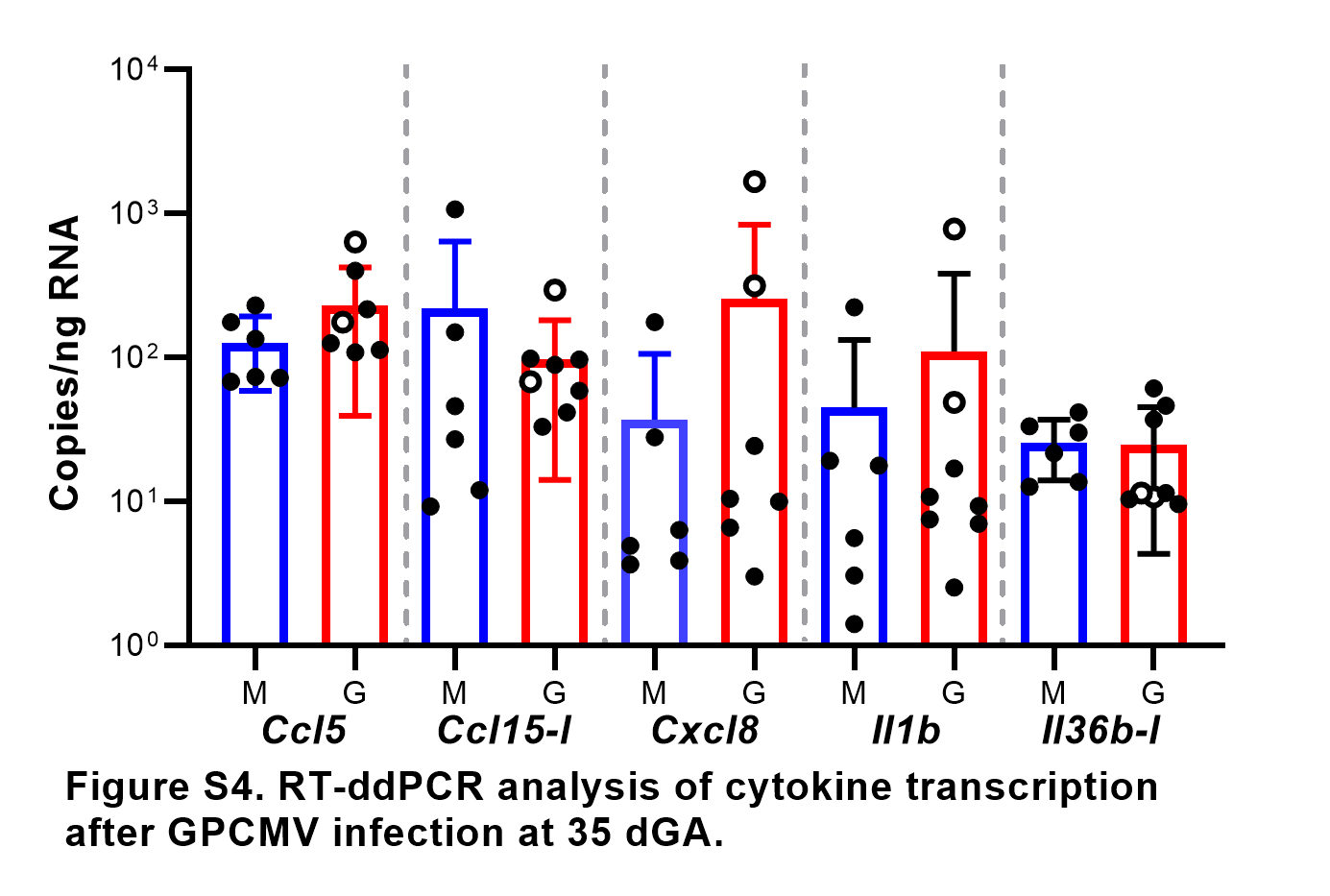

Supplement: Supplementary file 4 [file Image_4.tif]

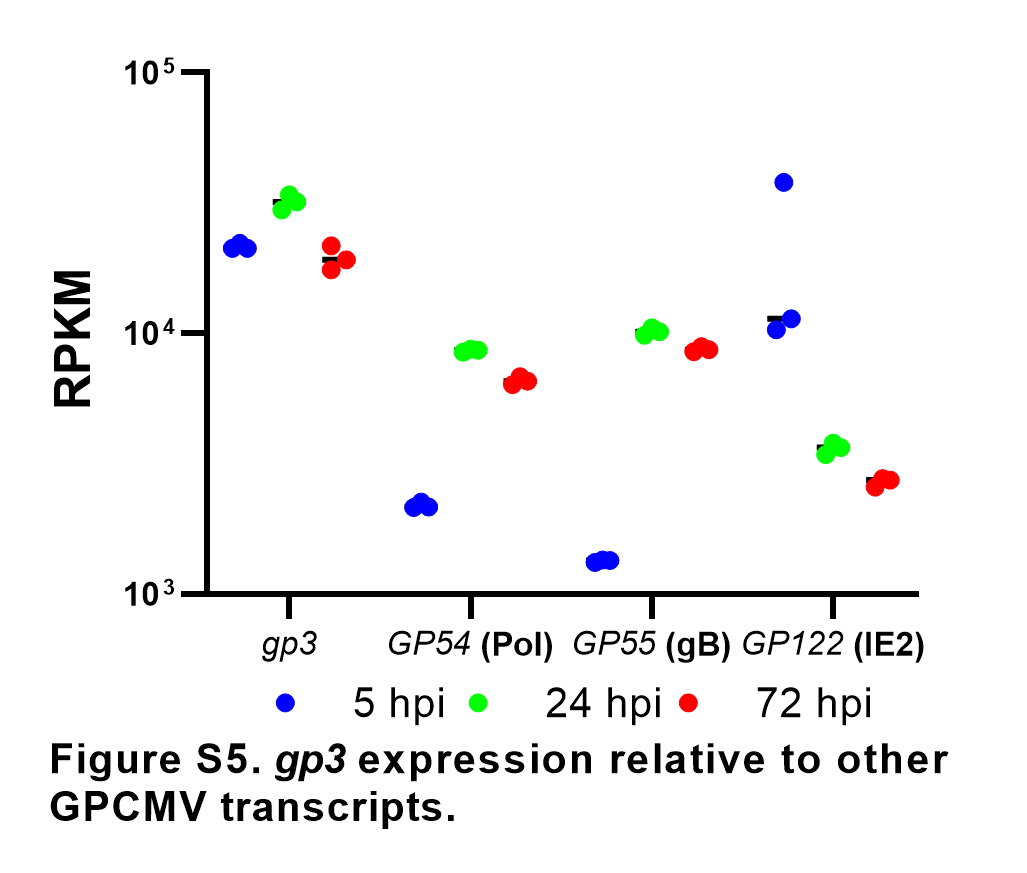

Supplement: Supplementary file 5 [file Image_5.tif]
