# Supplementary material for: The Placental Response to Guinea Pig Cytomegalovirus Depends Upon the Timing of Maternal Infection
Source: Front Immunol. 2021 Jun 15;12:686415. doi: 10.3389/fimmu.2021.686415 (PMC8239309; doi:10.3389/fimmu.2021.686415)
Supplement: Supplementary file 7 [file Table_2.docx]

| **Table S2.** Reverse transcriptase droplet digital PCR primer sets. | | | |
| --- | --- | --- | --- |
| **Gene** | **Locus** | **Forward (5′ → 3′)** | **Reverse (5′ → 3′)** |
| *Ccl5* | NM_001172927 | GGGAGGGCATGGATTCTATTAC | GACTCCCTTCCTCATTGCTAAG |
| *Ccl15-l* | XM_013152356.2 | CTGACTGCTGCTTCTCCTATAC | GCACCCACTACTTGTCCTAAA |
| *Cxcl8* | NM_001173399 | agaggactgagagccaagat | CACATCCACACAATGCAAGAAG |
| *Cxcl10* | XM_003477649 | GTGTCATGATCCTGAGCTGATAA | GGTGAGGAAAGAGTCAATAGCA |
| *Ido1* | XM_003464634.3 | GAAGTACCGCATAGACCAAGAG | CCTCAATCAGTTCCGTCAAGT |
| *Il1b* | NM_001172968.1 | CACTCCACGACATACAGCATAA | CACCACTTGTCGGTTCAGAT |
| *Il36b-l* | XM_023563014.1 | CAGTATTTCAGCCCTCCCATAG | GAGGGACATGGTGCAGAAA |
| *Tgtp1* | XM_005001267.3 | CCATTTCCTCAGGCAGTAACA | AGAGCATTAACAGGGTGATTGA |
| *Tlr8* | XM_003462892.2 | CAACTGTCCGAGGTGTTTCA | GTGGTGAAGGTTGCTCAGAT |
